# Ecological genomics of adaptation to unpredictability in experimental rotifer populations

**DOI:** 10.1038/s41598-019-56100-y

**Published:** 2019-12-23

**Authors:** Eva Tarazona, Christoph Hahn, Lluís Franch-Gras, Eduardo M. García-Roger, María José Carmona, Africa Gómez

**Affiliations:** 1 Institut Cavanilles de Biodiversitat i Biologia Evolutiva, Universitat de València, Valencia, Spain; 2 Department of Biological and Marine Sciences, University of Hull, Hull, United Kingdom; 3 Institute of Zoology, University of Graz, Graz, Austria

**Keywords:** Ecological genetics, Evolutionary ecology

## Abstract

Elucidating the genetic basis of phenotypic variation in response to different environments is key to understanding how populations evolve. Facultatively sexual rotifers can develop adaptive responses to fluctuating environments. In a previous evolution experiment, diapause-related traits changed rapidly in response to two selective regimes (predictable vs unpredictable) in laboratory populations of the rotifer *Brachionus plicatilis*. Here, we investigate the genomic basis of adaptation to environmental unpredictability in these experimental populations. We identified and genotyped genome-wide polymorphisms in 169 clones from both selective regimes after seven cycles of selection using genotyping by sequencing (GBS). Additionally, we used GBS data from the 270 field clones from which the laboratory populations were established. This GBS dataset was used to identify candidate SNPs under selection. A total of 76 SNPs showed divergent selection, three of which are candidates for being under selection in the particular unpredictable fluctuation pattern studied. Most of the remaining SNPs showed strong signals of adaptation to laboratory conditions. Furthermore, a genotype-phenotype association approach revealed five SNPs associated with two key life-history traits in the adaptation to unpredictability. Our results contribute to elucidating the genomic basis for adaptation to unpredictable environments and lay the groundwork for future evolution studies in rotifers.

## Introduction

Unraveling the genetic mechanisms underlying organisms’ phenotypic variation is essential to understanding how organisms adaptively respond to environmental fluctuations^1,2^. Anthropogenic effects and climate change have been identified as amplifiers of the variation in environmental fluctuations, and this effect is likely to further increase in the near future^3^. For that reason, adaptations to unpredictable fluctuations are particularly challenging to understand^4,5^. Research is needed to know whether organismal adaptive responses will allow them to cope with the upcoming increase in environmental unpredictability. Consequently, (1) understanding organisms’ evolutionary responses to unpredictability and (2) unraveling the molecular mechanisms and the genomic basis underlying these adaptations are key topics in evolutionary and conservation biology^6^.

Various adaptive responses have been described in organisms that inhabit fluctuating environments^4^. One of them is phenotypic plasticity, in which the same genotype produces different phenotypes depending on the environmental conditions^7^. Another mode of evolutionary response is adaptive tracking, in which natural selection acts recurrently on the standing heritable variation^8^. A third adaptive response is bet hedging, a life-history strategy that has been linked to unpredictable fluctuations^9^. This response occurs when a selected trait in a genotype increases the geometric mean of fitness at the cost of a decrease in the arithmetic mean, thus reducing fitness variance^10–12^. Despite the relevance of the ubiquitous challenge posed by environmental fluctuations and the diversity of adaptive responses displayed by organisms, empirical studies elucidating the genomic basis of these responses are still scarce.

Experimental evolution is a powerful tool that can be used to address diverse questions in evolutionary biology^13,14^ and explore the genomic basis of adaptation^15,16^ because it involves controlled laboratory conditions with a defined selective pressure. Typically, in this type of experiment, populations derived from a single ancestral genotype ─i.e., a genetically homogeneous population─ are exposed to different selective regimes^13,17^. However, an increasing number of studies have used a combination of several genotypes from one or more populations ─i.e., several ancestral genotypes or a polymorphic population─ to establish the experimental populations^18–20^. Evolutionary outcomes are expected to differ depending on whether the experimental populations are established from a single or from multiple genotypes. If the founding population is genetically monomorphic, adaptation to selective regimes could occur through the accumulation of *de novo* beneficial mutations. While not excluding this source of variation to fuel adaptive evolution, if the founding population is polymorphic ─and given the relative short-term scale of evolution experiments─ selection is expected to act mostly on the heritable standing variation^21–23^, which has been predicted to lead to rapid evolution in novel environments^24^.

Monogonont rotifers are common zooplankters of continental water bodies and are recognized as excellent model organisms for studies of experimental evolution (reviewed in Declerck & Papakostas^25^). These rotifers are facultatively sexual, combining asexual and sexual reproduction in their life cycle. Rotifer females reproduce asexually ─clonal proliferation─ until a specific environmental cue for sex initiation (e.g., the population density) induces some of these females to produce sexual daughters. These sexual females produce meiotic haploid eggs that develop into either haploid males, if they remain unfertilized, or into diploid diapausing eggs if they are fertilized^26–28^. Therefore, the product of sexual reproduction is a long-lived diapausing egg, so that sex is linked with diapause. Diapausing eggs are able to resist harsh environmental conditions such as desiccation, predation, freezing, etc.^28–30^. These eggs can remain viable in the sediments through at least several hydroperiods, forming diapausing egg banks, which are reservoirs of the past genetic diversity and adaptive phenotypic variation of the population^31^ and are essential for long-term population persistence^32^. In temperate climates, rotifer populations are typically ephemeral and recolonize the water column during the so-called planktonic growing season through the hatchlings of diapausing eggs, forming assemblages of clonal populations^32–34^. The rotifer species *Brachionus plicatilis* is a common inhabitant of salt lakes around the world^35,36^. These habitats are isolated from one another and are often characterized by strong seasonal fluctuations in the length of the growing season^37^ and can become unsuitable for periods of varying predictability^38^. These fluctuations are typical in the water bodies of the Mediterranean region^39^, which show a wide range of environmental predictability^40^. *B*. *plicatilis* populations in natural ponds are characterized by strong population structure and low levels of gene flow due to persistent founder effects^35,41,42^.

Using a set of nine *B*. *plicatilis* field populations from ponds covering a wide range of environmental predictability, Franch-Gras *et al*.^43^ found signatures of local adaptation to the degree of environmental predictability in two life-history traits (the timing of sex and the diapausing egg hatching fraction). In addition, high genetic variance related to these traits has been found in natural populations of *B*. *plicatilis*^43,44^. The ease of experimental culturing and the range of natural environmental unpredictability in rotifer habitats make rotifers ideal organisms for investigating the genomic basis of adaptation to environmental unpredictability. However, despite progress in identifying adaptive responses in rotifer life-history traits to environmental unpredictability, studies on the genetic basis underlying these responses are almost nonexistent. In a recent study using field rotifer populations, Franch-Gras *et al*.^45^ identified several candidate single-nucleotide polymorphisms (SNPs) as part of the genomic basis of local adaptation to fluctuating environments, which constitute a database for future genomic studies^45^.

In a previous evolution experiment, we used *B*. *plicatilis* to study the adaptation of life-history traits to unpredictable environments (for further details, see Tarazona *et al*.^46^). Six genetically identical laboratory populations were established by placing together 30 rotifer clones from each of the nine field populations representing a broad range of environmental predictability used by Franch-Gras *et al*.^43^. Since the rotifer clones from these populations were pooled to create the laboratory populations, hereafter we will refer to this field set as the “origin population”. Three laboratory populations (functioning as replicates) were randomly assigned to each selective regime (predictable vs unpredictable). These populations were studied during seven cycles of selection under a common garden experimental approach, tracking the evolution of two key diapause-related life-history traits: (1) the timing of sex ─as a proxy of the timing of diapause─ and (2) the diapausing egg hatching fraction ─inversely related to diapause duration. Rotifers showed rapid adaptation to a particular unpredictable fluctuation pattern of hydroperiod length, displaying a divergent response in those life-history traits in the populations subjected to the unpredictable regime with respect to those subjected to the predictable regime. Populations subjected to the unpredictable selective regime showed both lower hatching fractions of diapausing eggs and earlier sex initiation, suggesting that bet-hedging strategies evolve as an adaptation to environmental unpredictability in these organisms^46^. Although we used hydroperiod length fluctuation as the factor of predictability in our experimental design, it should be noted that several other factors (e.g., food, salinity, predation, etc.) might also affect the degree of predictability in the wild. Note that this experimental design involved testing for the effect of a particular pattern of unpredictable fluctuation in hydroperiod length, so we cannot generalize that the observed response is an adaptation to environmental unpredictability itself.

In this study, we used genotyping by sequencing (GBS)^47^ to identify the genomic signatures of selection. This technique relies on the use of restriction enzymes to reduce genome complexity and allows the identification and genotyping of single-nucleotide polymorphisms (SNPs) in individuals from different populations^48,49^. Thus, we identified and screened thousands of genomic markers by using GBS in laboratory *B*. *plicatilis* populations that evolved under predictable and unpredictable hydroperiod selective regimes^46^ to study the genomic basis of their adaptation. Additionally, to identify putative genes under selection and estimate the impact of genetic drift in the experimental populations, we used both an available *B*. *plicatilis* genome assembly from one clone from one of the field populations^45^ and genome-wide data obtained with the same methodology from the nine *B*. *plicatilis* field populations^45^ used to found the experimental laboratory populations. This large GBS dataset was used to identify candidate loci for diversifying selection under these selective regimes in the experimental rotifer populations. Moreover, the initial GBS information allowed us to estimate drift during the duration of the experiment for each particular laboratory population, as they can be compared with the origin population.

## Results

### GBS raw data and SNP calling and filtering

A total of 19 Gb of GBS raw data was obtained from the 169 clones from the six laboratory populations. A total of ca. 560 out of the ca. 915 million raw reads from the origin population plus the six laboratory populations were retained after quality filtering (see Material and Methods). After SNP calling and filtering, 6,107 SNPs were identified and genotyped (Table 1; vcf file: Supplementary Dataset S1) with an average coverage of 43.77 (7.02–109.25, median = 42.04). This set of SNPs was used in downstream analyses.

**Table 1:** Summary of SNP calling and filtering from the origin population plus the six laboratory populations of *B*. *plicatilis*.

Raw reads	915,507,331
Quality-filtered reads	506,614,399
SNP calling	
1- TASSEL pipeline	17,042
2- SNP filtering (1) and (2)	10,986
3- SNP filtering (3), (4) and (5)	6,424
4- SNP filtering (6)	6,107

Numbers in brackets refer to filtering criteria in VCFtools (see Methods section).

### Genetic structure

**Figure 1:**
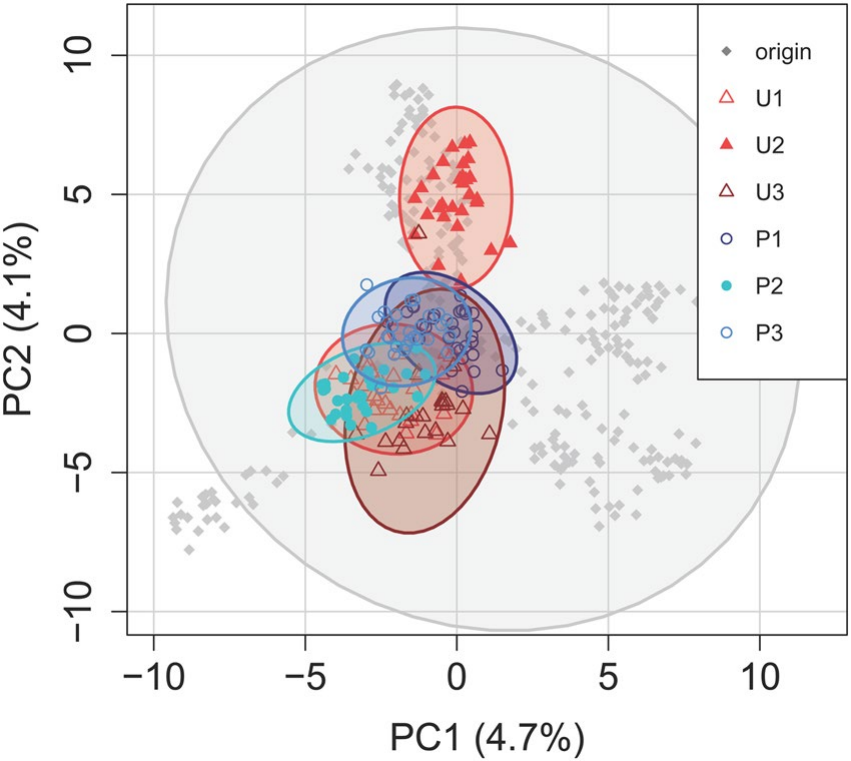
Principal component analysis (PCA) plot for the 6,107 SNPs of *B. plicatilis* clones from the six laboratory populations and the origin population. Dots indicate the location of the genotype of each clone in the space defined by the first (PC1; 4.7% variance explained) and second (PC2; 4.1% variance explained) principal components. Ellipsoids are the 95% confidence interval for the different populations. Symbol code: circles are populations under predictable regime; triangles are populations under unpredictable regime; diamonds are origin population. Colors represent different laboratory populations.

Clones from the origin population ─belonging to the nine field rotifer populations─ were widespread in the PCA ordination space, whereas most of the six laboratory populations were clustered in the center (Fig. 1). Pairwise *F*_*ST*_ values between the origin and the six laboratory populations were low, ranging from 0.004 to 0.025 (Table 2). These *F*_*ST*_ values were low in comparison with those of the field populations^42,45^, which suggests that genetic drift had little effect on the laboratory populations during the evolution experiment, although the unpredictable populations had slightly higher values, suggesting higher drift^50,51^. A mean *F*_*ST*_ value of 0.015 was found between selective regimes. Populations subjected to the predictable regime showed the lowest differentiation (Fig. 1 and Supplementary Fig. S1), with pairwise *F*_*ST*_ values ranging from 0.016 to 0.025 (Table 2). Populations under the unpredictable regime were more differentiated (Fig. 1 and Supplementary Fig. S1), with pairwise *F*_*ST*_ values ranging from 0.046 to 0.078. Expected heterozygosity (*H*_*e*_) ─i.e., genetic diversity─ was maintained in all laboratory populations (ranging from 0.17 to 0.21) after the evolution experiment, except for one of the populations that evolved under the unpredictable regime (U_2_), which had the lowest genetic diversity (for further details, see Supplementary Table S1). The values of genetic diversity were similar to those in the origin population (*He* = 0.21). Overall, all populations analyzed were broadly in Hardy-Weinberg equilibrium (*HWE*; Supplementary Fig. S2).

**Table 2:** Population pairwise fixation index (*F*_*ST*_) for the origin population and the six laboratory populations of *B*. *plicatilis* subjected to experimental evolution.

Population	P_1_	P_2_	P_3_	U_1_	U_2_	U_3_
P_2_	0.025					
P_3_	0.016	0.021				
U_1_	0.038	0.038	0.036			
U_2_	0.055	0.067	0.056	0.078		
U_3_	0.035	0.034	0.035	0.046	0.071	
Origin	0.005	0.008	0.004	0.013	0.015	0.010

*P*_*i*_: populations evolved under the predictable selective regime; *U*_*i*_: populations evolved under the unpredictable selective regime. The subindex *i* denotes replicate populations within each selective regime.

### Candidate SNPs under selection

**Figure 2:**
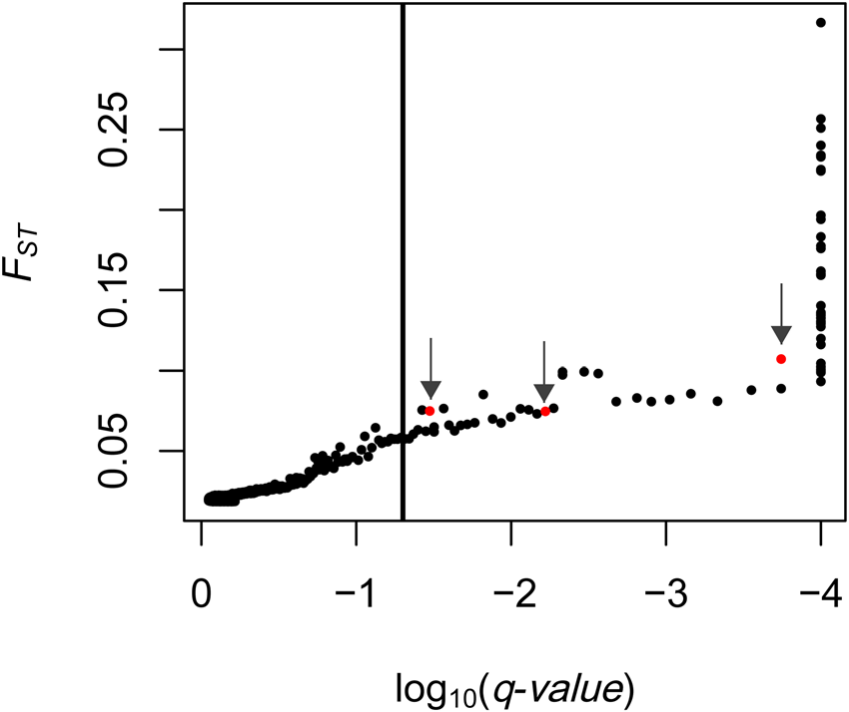
Identification of outlier loci putatively under selection in *B. plicatilis* populations using BayeScan analysis for the 6,107 genotyped SNPs. The marker-specific *FST* is plotted against the decision factor to determine selection in base-10 log scale log_10_(*q*-value) using a false discovery rate (FDR) of 0.05. Markers on the right side of the vertical line (*q* < 0.05) are considered outliers. This analysis included the origin population and the populations evolved under two selective regimes (predictable vs unpredictable). Each dot represents a SNP. Arrows point to the SNPs identified as being putatively under selection between selective regimes (see Supplementary Fig. S3).

A total of 76 candidate SNPs under selection were identified by BayeScan (BS) analysis with prior odds (PO) = 10 among the origin population and the two selective regimes (BS1 analysis; Fig. 2). These candidate SNPs were on 45 scaffolds showing evidence of being under selection. Three of the candidate SNPs under selection were identified in the comparison of the two selective regimes (BS2 analysis with PO = 10; red dots in Fig. 2; Supplementary Fig. S3). These three unlinked candidate SNPs (S4644_2726, S9060_3689 and S78024_5745) had undergone changes in allele frequencies in the populations under the unpredictable regime, diverging from the origin population (Table 3). This divergence was not found in the predictable populations, which had similar allelic frequencies to the origin population. Most of the remaining SNPs identified in the BS1 analysis showed parallel changes in all six laboratory populations, suggesting adaptation to laboratory conditions.

**Table 3:** Allelic frequencies in the origin population and the six laboratory populations of *B*. *plicatilis* for the three SNPs candidate to be under selection between both selective regimes.

Candidate SNP	Origin	*P*_*1*_	*P*_2_	*P*_3_	*U*_1_	*U*_2_	*U*_3_
S4644_2726	0.688	0.500	0.569	0.750	0.867	1.000	1.000
S9060_3689	0.924	0.914	0.724	0.857	1.000	1.000	1.000
S78024_5745	0.775	0.788	0.760	0.780	1.000	1.000	1.000

*P*_*i*_: populations under the predictable selective regime; *U*_*i*_: populations under the unpredictable selective regime. The subindex *i* denotes replicate population within selective regime.

### Genome-wide association analysis

**Figure 3:**
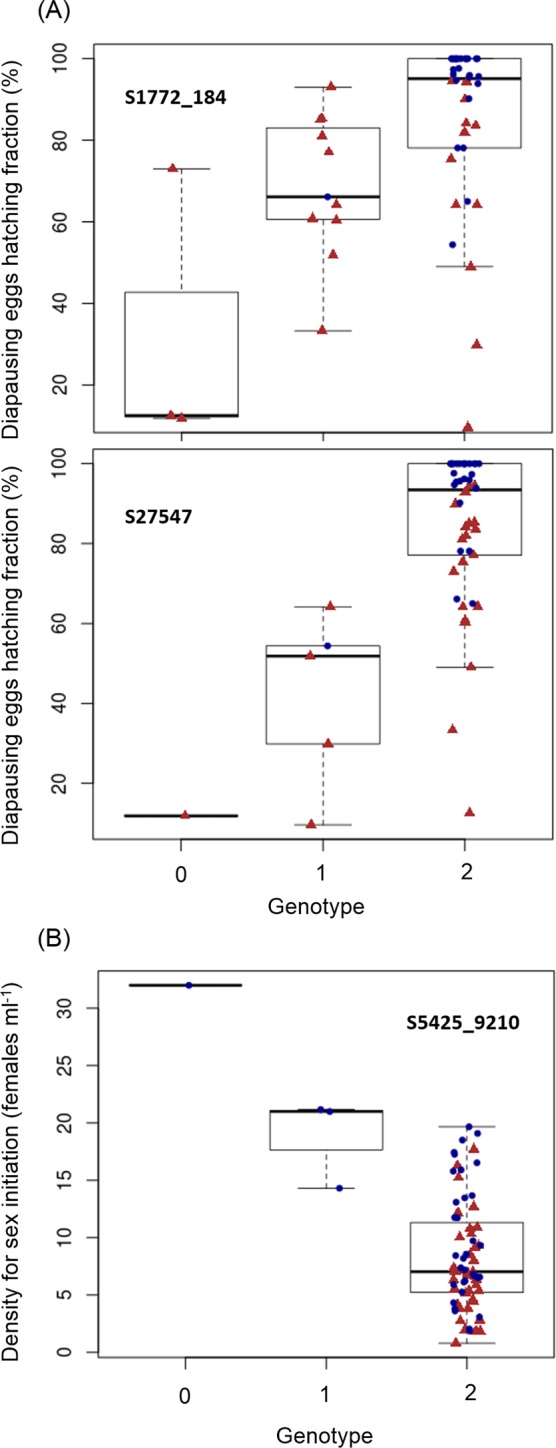
Genotype-phenotype association patterns for two life-history traits of *B. plicatilis*: hatching fraction and timing of sex. Box-and-whisker plot of the genotypes of SNPs that are most significantly associated with (**A**) diapausing egg hatching fraction (SNP S1772_184 and three SNPs associated to the scaffold S27547), and (**B**) timing of sex, estimated as the density for sex initiation (SNP S5425_9210). Dots (jittered in the x direction) represent each clone, those evolved under the predictable regime are represented by blue circles, whereas clones evolved under the unpredictable one by red triangles. Phenotypic data obtained from Tarazona *et al*.^46^.

A genotype-phenotype association analysis, using the subset of genotyped clones for which life-history traits were available, revealed a significant association between five SNPs and the assessed traits at a significance level of 0.05/N (N = 52 and 76 clones for the diapausing egg hatching fraction and the timing of sex, respectively). Four SNPs in two scaffolds (S1772_184, S27547_4262, S27547_4267 and S27547_4271) were associated with the hatching fraction (Fig. 3A and Supplementary Fig. S4), with an adjusted *p*-value = 0.0201 for SNP S1772_184 and *p*-value = 0.0015 for the other three SNPs. The three SNPs in scaffold S27547 were 9 bases apart. One SNP (S5425_9210) was associated with the timing of sex (Fig. 3B and Supplementary Fig. S4) with an adjusted *p*-value = 0.0288.

### Identification of candidate genes under selection

The 76 SNPs identified by BS1 as candidates of being under positive selection were located within 43 genes, 53.5% of which had putative associated functions (see Supplementary Table S2). Following Franch-Gras *et al*.’s^45^ criteria, additional analyses were performed using just those genes including a SNP in their coding region (i.e., genes at a 0 Kb distance). GO enrichment analysis showed that they were enriched at high levels (i.e., general GO terms), such as the regulation of biological processes (GO:0050789), cellular processes (GO:0009987), single-organism processes (GO:0044699), the regulation of cellular processes (GO:0050794), binding (GO:0005488), and biological regulation (GO:0065007). The three candidate SNPs to be under selection between selective regimes were located in three genes: *Ribosomal S6 kinase alpha-*1*-isoform X1*, *RNA-binding single-stranded-interacting 3* and *Midasin*.

Those SNPs identified as putatively associated with the hatching fraction of diapausing eggs in the genotype-phenotype analysis were located in two genes, but only one had an associated function: the gene *kelch-like ECH-associated 1*. No gene was found for the candidate SNP associated with the timing of sex.

## Discussion

We identified genomic signatures underlying rapid adaptation in rotifer populations under two contrasting laboratory selective regimes (predictable vs unpredictable) using an experimental evolution approach and genome-wide genotyping. Three out of 45 scaffolds had signatures of divergent selection in response to an unpredictable environmental fluctuation pattern. Most of the remaining scaffolds with signatures of directional selection experienced parallel allele changes in all six laboratory populations, suggesting adaptation to laboratory environmental conditions. These populations showed signatures of adaptation to the experimental unpredictable environment in their life-history traits (i.e., the timing of sex and diapausing egg hatching fraction)^46^. In addition, the genome-wide association analysis performed here revealed three scaffolds associated with both life-history traits. Overall, our results indicate that there is substantial genetic diversity underlying the adaptation to environmental unpredictability in the original rotifer populations.

We found that three SNPs located in three different genes showed signatures of positive selection in response to the unpredictable environmental fluctuation pattern studied. The SNP S9060_3689 was located within the gene *RNA-binding single-stranded-interacting 3* (RBMS3), a member of the family of c-myc single-strand binding protein (MSSP) genes. These genes are involved in DNA replication, gene transcription, cell cycle progression and apoptosis in humans^52^ and regulate one of the major pathways promoting chondrogenesis in zebrafish^53^. The SNP S4644_2726 is located within the *ribosomal S6 kinase alpha-1 isoform X1* gene and is involved in controlling growth and differentiation in human cells^54^. Finally, the SNP S78024_5745 was located in the *Midasin* gene, which encodes a nuclear chaperone involved in the assembly/disassembly of complex macromolecules in yeast^55^ and has been shown to be essential for the normal progression of female gametogenesis in *Arabidopsis thaliana*^56^. Regarding the genotype-phenotype association analysis, four SNPs putatively associated with the diapausing egg hatching fraction were found. Three of them were fully linked and located within the same gene ─*kelch-like ECH-associated 1*─ which is involved in responses to oxidative stress^57–59^. This gene has been described in several groups, including fish^60^, insects^61^, and mammals^62^.

In general, the experimental populations showed weak genetic drift, as evidenced by the low genome-wide pairwise *F*_*ST*_ values (*F*_*ST*_ < 0.1; mean *F*_*ST*_ between selective regimes was 0.015) when compared to those of the original field populations (*F*_*ST*_ = 0.25 in Montero-Pau *et al*.^42^; *F*_*ST*_ = 0.18 in Franch-Gras *et al*.^45^). All but one population maintained their genetic diversity throughout the experiment. Nevertheless, the negligible loss of genetic diversity (*H*_*e*_) and the minor variation in *F*_*ST*_ values among populations suggest that genetic drift was of minor importance in comparison to selection^13,16,63,64^.

A strong signal of adaptation to laboratory conditions during the selection experiment was found. This was derived from the number of candidate SNPs under selection with parallel allelic frequency changes identified in all six laboratory populations regardless of the selective regime. These results were not unexpected^65,66^ given that (1) the laboratory populations were derived directly from the field and (2) the laboratory conditions other than the selective regime were identical and consistent across the selective regimes in the evolution experiment (e.g., temperature, salinity, food type and availability, light, length of dry phase, etc.). This contrasts with the different and highly variable conditions experienced by the field populations^66^ from which the origin population was derived^43^. The selective regimes mimicked the extreme patterns of selection imposed by the environmental unpredictability in the hydroperiod length to which *B*. *plicatilis* natural populations are exposed^40^. We focused on the unpredictability of the hydroperiod length, as it is expected to exert a strong selection pressure on ecologically relevant traits of rotifer life history, such as the diapausing egg hatching fraction and the timing of sex^37,43,67^. However, unpredictability is likely generated by other environmental factors that might also be associated with hydroperiod length in natural populations (e.g., salinity, temperature, food, antagonists, etc.) and could be expected to create additional selective pressures that were not explored here.

Genomic signatures of rapid adaptation in laboratory populations through experimental evolution have been identified, supporting previous results showing rapid phenotypic divergence in rotifer populations, which evolved divergent life-history traits over a short time span^46^. Although other experimental evolution studies have shown adaptation to a range of selective pressures in rotifers^25,68^ and other well-known cyclical parthenogens, such as *Daphnia*^69,70^, the genomic basis has been little explored. Genomic signatures of adaptation to environmental anthropogenic stressors were found in *D*. *magna* using an experimental evolution approach^71^; however, the genomic basis of adaptation to environmental unpredictability is essentially unknown. To the best of our knowledge, only a recent study involving rotifer field populations identified genomic signatures of local adaptation to unpredictable environments^45^. Nonetheless, the genetic variants that could be key in the adaptation to unpredictable environments are not shared between the two studies. It appears unlikely that only three genes underlie the genetic architecture of the life-history trait response to environmental unpredictability, given that such traits are likely to be affected by large numbers of genes^72^. In this regard, it is worth noting that several limitations constrain our results: (1) genes across the genome with small effects may not be detected as significant outliers given the power of evolution experiments, (2) we use a sample of SNPs on a draft genome, (3) the experiment is not designed to detect elements of adaptation due to idiosyncratic changes in each population, (4) selective pressures associated with habitat unpredictability in the field might differ from those in our experimental evolution approach^73^, and (5) we recognize that the adaptation was to a particular pattern of unpredictable environment rather than to environmental unpredictability itself. To assess adaptation to environmental unpredictability (and its genomic basis), several replicated patterns of unpredictability would have to be included (of course implying greater complexity in the logistics of the experiment). These limitations could explain the discrepancies found between the results reported by Franch-Gras *et al*.^45^ and those of the present study.

The recent availability of a draft genome for *B*. *plicatilis*^45^ allowed us to call SNPs more confidently and to identify putative genes under selection. Although tentative functionality has been assigned to most of the genes containing those SNPs (53.3%), the lack of functional annotation for many of the genes of *B*. *plicatilis* precludes the understanding of the mechanisms under selection in this rotifer species.

In conclusion, we have shown genomic evidence of rapid adaptation in experimental populations of the rotifer *B*. *plicatilis*, building on previous results for ecologically relevant traits. Candidate SNPs under selection from a particular unpredictable fluctuation pattern were identified, some of them associated with key life-history traits in the life cycle of this rotifer species. Given that these responses allow monogonont rotifers to adapt to unpredictable environments, our results can serve as a basis for future studies focused on adaptation. In addition, the rotifer populations showed strong genomic signals of adaptation to laboratory conditions. Furthermore, this study shows the potential of using monogonont rotifer populations to evaluate signatures of adaptation in short-term experiments. Nonetheless, (1) a better understanding of the *B*. *plicatilis* genome and transcriptome and (2) experimental validation of the predicted genes are still necessary to elucidate the genomic basis of rotifer adaptation to unpredictable environments.

## Material and Methods

### Experimental laboratory populations and study clones

**Figure 4:**
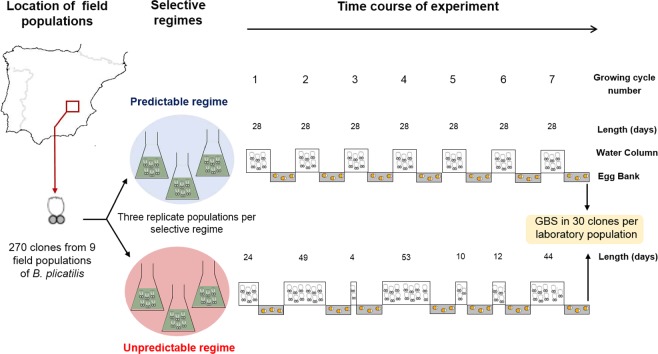
Schematic experimental evolution design. Modified from Tarazona *et al*.^46^.

The populations analyzed were obtained from the last cycle of the evolution experiment described in Tarazona *et al*.^46^, in which the effects of environmental unpredictability on diapause-related traits in rotifers were tested. Briefly, in that study, six genetically identical multiclonal laboratory populations of the rotifer *B*. *plicatilis* were founded by placing together asexual females from each of 30 clonal lines from nine Spanish Mediterranean salt ponds and lakes (a total of 270 clones^43^). These populations were subjected to two contrasting selective regimes (predictable vs unpredictable) during seven growing cycles of selection mimicking planktonic growing seasons. These growing cycles were interrupted by periods of habitat unsuitability ─dry periods─ (see Fig. 4). Three laboratory populations (functioning as replicates) were randomly assigned to the predictable regime, characterized by a regular hydroperiod length, and the other three were assigned to the unpredictable regime, characterized by a random hydroperiod length. The end of each growing cycle in both selective regimes was simulated by filtering the cultures and allowing the filtrate, containing diapausing eggs, to dry. Only the diapausing eggs were able to survive these drying events and restart the following growing cycle through the hatchlings after a period of diapause. Note that the unpredictable fluctuation pattern (i.e., the particular sequence of different growing cycle lengths) was the same for the three replicate populations, so we tested the effect of this particular pattern of fluctuation and not any unpredictable fluctuation in the growing cycle length. Notably, a few growing cycles in the unpredictable regime were so short that few or no new diapausing eggs were produced, so populations relied on hatchlings from the accumulated egg bank. These cycles impose a strong selective pressure in our experimental design. Precisely, the fact that within the randomly established sequence, there was an extremely short cycle in which the production of a new cohort of diapausing eggs is not possible allows the assessment of whether (1) the persistence of rotifer populations depends on the existence of the egg bank and (2) the rotifers experience a truly adverse cycle that they cannot anticipate. By including several replicate experimental populations within each selective regime in the experimental evolution design, it was possible to evaluate their independent evolutionary trajectories. The experiment lasted 392 days ─including growing cycles plus diapause periods (for further details, see Tarazona *et al*.^46^).

After the seventh cycle of selection, a total of 30 clones from each of the six laboratory populations were used to found clonal lines (180 clones in total) to carry out the DNA extraction. It is worth noting that 10 of these clones were also used to perform the life-history trait assays described in Tarazona *et al*.^46^.

### DNA extraction

To obtain sufficient biomass for DNA extractions, each individual experimental clone was grown under standard culture conditions (12 g L^−1^ saline water, maintained at 20 °C, and fed the microalga *Tetraselmis suecica*) in 1.5 L plastic bottles for 15 days, starting from a low-density stock culture. On day 15, when an abundance of more than 15,000 individuals was reached in each bottle, the cultures were filtered out through a 30-µm Nytal mesh sieve. The retained rotifers were released and kept in saline water (12 g L^−1^) for 24 hours to purge their digestive tract and minimize contamination with microalgal DNA. Thereafter, the rotifers were retained on a 30-µm Nytal mesh sieve, which was frozen in liquid nitrogen. DNA extraction was performed on the frozen rotifer biomass using a JETFLEX Genomic DNA purification kit (GENOMED, Löhne, Germany) following the manufacturer’s instructions. DNA quality was assessed on a 1% agarose gel, and the quantification of DNA was performed with a Qubit 2.0 fluorometer (Life Technologies, Thermo Fisher Scientific Inc.). A total of 169 clones were obtained with a sufficient DNA quality and concentration (>20 ng/µl of DNA) to apply the genotyping-by-sequencing protocol.

### Genotyping by sequencing (GBS)

GBS libraries were prepared and sequenced at the Institute for Genomic Diversity (IGD, Ithaca, NY, USA) following the protocol described by Elshire *et al*.^47^. In brief, (1) DNA samples were digested with the restriction enzyme *ApeKI* (GC[A-T]GC), (2) GBS libraries were constructed with unique barcodes for each clone, and (3) after pooling the samples, sequencing was carried out using an Illumina HiSeq. 2000/2500 (100 bp, single-end).

### SNP calling and filtering

Raw sequence data quality was analyzed using FastQC version 0.11.5 (www.bioinformatics.babraham.ac.uk/projects/fastqc/). Additionally, the published GBS data from the 270 clones used as founders of the experimental laboratory populations (hereafter origin population) were downloaded and employed for comparison (accession number SRP151997)^45^. SNPs were called from the raw DNA reads using the SNP calling v.2 pipeline as implemented in TASSEL 5^74^. All reads were trimmed to 64 bp, and identical reads were collapsed into tags. Thereafter, these tags were aligned against a draft assembly of the *B*. *plicatilis* genome of 108 Mb (accession number REGN00000000)^45^ using the Burrows-Wheeler alignment tool (BWA)^75^, and the SNPs were called from the aligned tags. The default parameters from the TASSEL-GBS pipeline were used with some modifications intended to apply a more conservative approach: the minimum length of an aligned base pair (-aLen 30 instead of default 0) and the minimum locus coverage (-mnLCov 0.8 instead of default 0.1).

The set of SNPs was filtered by quality using scripts modified from Franch-Gras *et al*.^45^ (see Supplementary Dataset 3 in Franch-Gras *et al*.^45^) and VCFtools^76^ to construct the final “individual (clone) x genotype” matrix. The following parameters were set: (1) a minimum coverage of six reads to call a genotype, (2) a minimum of 50% of the individuals of each population genotyped, (3) a minimum allele frequency (–maf) higher than 1%, (4) only two alleles present (–min-alleles,–max-alleles), (5) average read depth <150 (–max-meanDP), and (6) heterozygotes in each SNP < 60%.

### Data analysis

#### Genetic structure

To assess the genome-wide genetic variation and differentiation, a principal component analysis (PCA) was performed on individual clone genotypes of all populations using the “adegenet” package^77^ in R^78^ v. 3.2.2. To evaluate between-population differentiation and the effects of drift, the fixation index (*F*_*ST*_) for each pairwise comparison was estimated. Additionally, the *F*_*ST*_ value between selective regimes was calculated by grouping the three replicates (i.e., the three populations) under each selective regime. Other basic statistics, such as the expected heterozygosity (*H*_*e*_), observed heterozygosity (*H*_*o*_), and inbreeding coefficient (*F*_*IS*_), for the origin and six experimental laboratory populations were estimated. *F*_*ST*_ values, *H*_*e*_, *H*_*o*_ and *F*_*IS*_ were estimated using the R package “hierfstat”^79^. The function *pairwise*.*fst* within this package computes Nei’s pairwise *F*_*ST*_ between pairs of populations^80^. Hardy-Weinberg equilibrium (*HWE*) was tested using VCFtools for each population. File format conversions were performed using PGDSpider^81^ and Plink^82^ v.1.9.

#### Candidate SNPs under selection

Putative selected loci were identified in BayeScan^83^ v.2.1. This genome-scan method identifies markers that show stronger divergence patterns between groups than would be expected under neutral genetic processes. Moreover, it estimates the posterior probability that a given SNP is affected by selection. This *F*_*ST*_-based method is a differentiation method that is robust to confounding demographic processes^84,85^ and has a low number of false positives^86^ relative to other methods. We carried out two BayeScan analyses, BS1 and BS2, using prior odds (PO) of 100 and 10 and a false discovery rate (FDR) of 0.05. PO refers to the ratio of posterior probabilities, which indicates the likelihood of the selection model in comparison to that of the neutral model^87,88^. Therefore, here, the selection model must be 10 or 100 times more likely than the neutral model for a locus to be considered as a candidate of being under selection. In BS1, the following groupings of populations were established: (1) origin populations, (2) the three replicate populations that evolved under the predictable regime (hereafter predictable populations), and (3) the three replicate populations that evolved under the unpredictable regime (hereafter unpredictable populations). In BS2, to assess the assignment of candidate SNPs to each selective regime (predictable vs unpredictable), we used two groupings: (1) predictable populations and (2) unpredictable populations. This design allowed us to observe parallel adaptive responses in replicate populations to obtain relatively robust results, but it did not allow us to identify idiosyncratic ─population-specific─ changes in the populations.

#### Genome-wide association analysis (GWAS)

Genotype-phenotype association analysis was performed for the two life-history traits ─timing of sex and hatching fraction of diapausing eggs─ obtained for each clone in our previous experimental evolution study^46^. We included clones with both phenotypic and genotypic data available (52 and 76 clones for hatching fraction and timing of sex, respectively; Supplementary Dataset S2). In this dataset, the timing of sex is expressed as females mL^−1^, and the hatching fraction of diapausing eggs is expressed as the percentage of hatching eggs. Phenotype-genotype association analysis was performed by running linear models using Plink version 1.9^82^ (Supplementary Method S1). The SNP dataset was filtered for a minimum allele frequency lower than 0.01. We implemented a stratified analysis controlling by population assignment and adjusting *p*-values with the Bonferroni correction (<0.05). Linkage disequilibrium (LD) was also assessed for candidate SNPs associated with both phenotypic traits using Plink.

#### Identification of candidate genes under selection

Gene functions were retrieved from the current annotation of the *B*. *plicatilis* genome^45^. Genes putatively associated with the candidate SNPs identified with BayeScan and those identified with GWAS were identified in the flanking regions of 0, 2.5 and 5 kbp upstream and downstream of the focal SNPs using BEDtools^89^. Additionally, a gene ontology (GO) enrichment analysis (Fisher’s exact test, two-tailed, false discovery rate <0.05) was performed for those genes in which candidate SNPs under selection were found using Blast2GO^90^.

## Supplementary information

Supplementary information

Supplementary Dataset S2

Supplementary Dataset S1

## Data Availability

The sequences used in this study were deposited in GenBank (BIOproject ID: PRJNA503132). GBS raw sequences were deposited in the ENA (Accession Number SRR8144248).

## References

[1] Storz JF (2005). Using genome scans of DNA polymorphism to infer adaptive population divergence. Mol. Ecol..

[2] Stinchcombe JR, Hoekstra HE (2008). Combining population genomics and quantitative genetics: finding the genes underlying ecologically important traits. Heredity.

[3] IPCC. Climate change 2013. The Physical Science Basis. Contribution of Working Group I to the Fifth Assessment Report of the Intergovernmental Panel on Climate Change (Cambridge University Press, 2013).

[4] Simons AM (2011). Modes of response to environmental change and the elusive empirical evidence for bet hedging. Proc. R. Soc. B Biol. Sci..

[5] Botero CA, Weissing FJ, Wright J, Rubenstein DR (2015). Evolutionary tipping points in the capacity to adapt to environmental change. Proc. Natl. Acad. Sci. USA.

[6] Harrisson KA, Pavlova A, Telonis-Scott M, Sunnucks P (2014). Using genomics to characterize evolutionary potential for conservation of wild populations. Evol. Appl..

[7] Reed TE, Waples RS, Schindler DE, Hard JJ, Kinnison MT (2010). Phenotypic plasticity and population viability: the importance of environmental predictability. Proc. R. Soc. B Biol. Sci..

[8] Tufto J (2015). Genetic evolution, plasticity, and bet-hedging as adaptive responses to temporally autocorrelated fluctuating selection: A quantitative genetic model. Evolution.

[9] Ripa J, Olofsson H, Jonzén N (2010). What is bet-hedging, really?. Proc. R. Soc. B Biol. Sci..

[10] Seger J, Brockmann HJ (1987). What is bet-hedging?. Oxford Surveys in Evolutionary Biology.

[11] Childs DZ, Metcalf CJE, Rees M (2010). Evolutionary bet hedging in the real world: empirical evidence and challenges revealed by plants. Proc. R Soc. Lond. B Biol. Sci..

[12] Schreiber SJ (2015). Unifying within- and between-generation bet-hedging theories: An ode to J. H. Gillespie. Am. Nat..

[13] Kawecki TJ (2012). Experimental evolution. Trends Ecol. Evol..

[14] Kang L, Agarwal DD, Rashkovetsky E, Korol AB, Michalak P (2016). Rapid genomic changes in *Drosophila melanogaster* adapting to desiccation stress in an experimental evolution system. BMC Genomics.

[15] Barrett RD, Hoekstra HE (2011). Molecular spandrels: tests of adaptation at the genetic level. Nat. Rev. Genet..

[16] Matos M (2015). History, chance and selection during phenotypic and genomic experimental evolution: replaying the tape of life at different levels. Front.Genet..

[17] Teotónio H, Estes S, Phillips PC, Baer CF (2017). Experimental Evolution with *Caenorhabditis* Nematodes. Genetics.

[18] Graham JK, Smith ML, Simons AM (2014). Experimental evolution of bet hedging under manipulated environmental uncertainty in *Neurospora crassa*. Proc. R Soc. Lond. B Biol. Sci..

[19] Sikkink KL, Reynolds RM, Ituarte CM, Cresko WA, Phillips PC (2014). Rapid evolution of phenotypic plasticity and shifting thresholds of genetic assimilation in the nematode *Caenorhabditis remanei*. G3 (Bethesda).

[20] Declerck SAJ (2015). Rapid adaptation of herbivore consumers to nutrient limitation: eco-evolutionary feedbacks to population demography and resource control. Ecol. Lett..

[21] Barrett RD, Schluter D (2008). Adaptation from standing genetic variation. Trends Ecol. Evol..

[22] Hohenlohe PA (2010). Population genomics of parallel adaptation in threespine stickleback using sequenced RAD tags. PLoS Genet..

[23] Schlötterer C, Kofler R, Versace E, Tobler R, Franssen SU (2015). Combining experimental evolution with next-generation sequencing: a powerful tool to study adaptation from standing genetic variation. Heredity.

[24] Hermisson J, Pennings PS (2005). Soft Sweeps. Molecular population genetics of adaptation from standing genetic variation. Genetics.

[25] Declerck SAJ, Papakostas S (2017). Monogonont rotifers as model systems for the study of micro-evolutionary adaptation and its ecoevolutionary implications. Hydrobiologia.

[26] Pourriot R, Snell TW (1983). Resting eggs in rotifers. Hydrobiologia.

[27] Gilbert JJ (2002). Endogenous regulation of environmentally induced sexuality in a rotifer; a multigenerational parental effect induced by fertilisation. Freshwater Biol..

[28] Schröder T (2005). Diapause in monogonont rotifers. Hydrobiologia.

[29] Hairston, N. G. & Fox, J. A. (2009). Egg banks in *Encyclopedia of Inland Waters* (ed. Likens, G. E.) 659–666 (Academic Press, 2009).

[30] Radzikowski J (2013). Resistance of dormant stages of planktonic invertebrates to adverse environmental conditions. J. Plankton Res..

[31] Hairston NG (1996). Zooplankton egg banks as biotic reservoirs in changing environments. Limnol. Oceanogr..

[32] García-Roger EM, Carmona MJ, Serra M (2006). Patterns in rotifer diapausing egg banks: density and viability. J. Exp. Mar. Bio. Ecol..

[33] Gómez A, Carvalho GR (2000). Sex, parthenogenesis and genetic structure of rotifers: microsatellite analysis of contemporary and resting egg bank populations. Mol. Ecol..

[34] Kotani T, Ozaki M, Matsuoka K, Snell TW, Hagiwara A (2001). Reproductive isolation among geographically and temporally isolated marine *Brachionus* strains. Hydrobiologia.

[35] Gómez A, Serra M, Carvalho GR, Lunt DH (2002). Speciation in ancient cryptic species complexes: evidence from the molecular phylogeny of *Brachionus plicatilis* (Rotifera). Evolution.

[36] Mills S (2017). Fifteen species in one: deciphering the *Brachionus plicatilis* species complex (Rotifera, Monogononta) through DNA taxonomy. Hydrobiologia.

[37] García-Roger EM, Serra M, Carmona MJ (2014). Bet-hedging in diapausing egg hatching of temporary rotifer populations: A review of models and new insights. Int. Rev. Hydrobiol..

[38] Ortells R, Snell TW, Gómez A, Serra M (2000). Patterns of genetic differentiation in resting egg banks of a rotifer species complex in Spain. Arch.Hydrobiol..

[39] Blondel, J., Aronson, J., Bodiou, J-Y. & Boeuf, G. *The Mediterranean Region*. *Biological diversity in space and time* (Oxford University Press, 2010).

[40] Franch-Gras L, García-Roger EM, Carmona MJ, Serra M (2017). Quantifying unpredictability: a multiple model approach for Mediterranean ponds by using satellite imagery. PLoS ONE.

[41] Gómez A, Montero‐Pau J, Lunt D (2007). Persistent genetic signatures of colonization in *Brachionus manjavacas* rotifers in the Iberian Peninsula. Mol. Ecol..

[42] Montero-Pau J, Serra M, Gómez A (2017). Diapausing egg banks, lake size, and genetic diversity in the rotifer *Brachionus plicatilis* Müller (Rotifera, Monogononta). Hydrobiologia.

[43] Franch-Gras L, García-Roger EM, Serra M, Carmona MJ (2017). Adaptation in response to environmental unpredictability. Proc. R. Soc. B Biol. Sci..

[44] Campillo S, García-Roger EM, Carmona MJ, Serra M (2009). Selection on life-history traits and genetic population divergence in rotifers. J. Evol. Biol..

[45] Franch-Gras L (2018). Genomic signatures of local adaptation to the degree of environmental predictability in rotifers. Sci. Rep..

[46] Tarazona E, García‐Roger EM, Carmona MJ (2017). Experimental evolution of bet hedging in rotifer diapause traits as a response to environmental unpredictability. Oikos.

[47] Elshire RJ (2011). A robust, simple genotyping-by-sequencing (GBS) approach for high diversity species. PLoS ONE.

[48] Davey JW (2011). Genome-wide genetic marker discovery and genotyping using next-generation sequencing. Nat. Rev. Genet..

[49] Narum SR, Buerkle CA, Davey JW, Miller MR, Hohenlohe PA (2013). Genotyping‐by‐sequencing in ecological and conservation genomics. Mol. Ecol..

[50] Bhatia G, Patterson N, Sankararaman S, Price AL (2013). Estimating and interpreting FST: The impact of rare variants. Genome Res..

[51] Tills D (1997). The Use of the Fsτ Statistic of Wright for Estimating the Effects of Genetic Drift, Selection and Migration in Populations, with Special Reference to Ireland. Hum. Hered..

[52] Penkov D (2000). Cloning of a human gene closely related to the genes coding for the cmyc single-strand binding proteins. Gene.

[53] Jayasena CS, Bronner ME (2012). Rbms3 functions in craniofacial development by posttranscriptionally modulating TGF-β signaling. J. Cell Biol..

[54] Moller DE, Xia CH, Tang W, Zhu AX, Jakubowski M (1994). Human rsk isoforms: cloning and characterization of tissue-specific expression. Am. J. Physiol..

[55] Garbarino JE, Gibbons IR (2002). Expression and genomic analysis of midasin, a novel and highly conserved AAA protein distantly related to dynein. BMC Genomics.

[56] Chantha S-C, Gray-Mitsumune M, Houde J, Matton DP (2010). The *MIDASIN* and *NOTCHLESS* genes are essential for female gametophyte development in *Arabidopsis thaliana*. Physiol. Mo. Biol. Plants.

[57] Itoh K (1999). Keap1 represses nuclear activation of antioxidant responsive elements by Nrf2 through binding to the amino-terminal Neh2 domain. Genes Dev..

[58] Dhakshinamoorthy S, Jaiswal AK (2001). Functional characterization and role of INrf2 in antioxidant response element-mediated expression and antioxidant induction of NAD(P)H: quinone oxidoreductase 1 gene. Oncogene.

[59] Hayes JD, McMahon M (2009). NRF2 and KEAP1 mutations: permanent activation of an adaptive response in cancer. Trends Biochem. Sci..

[60] Penglase S (2015). Diet affects the redox system in developing Atlantic cod (*Gadus morhua*) larvae. Redox Biol..

[61] Kelso RJ, Hudson AM, Cooley L (2002). Drosophila Kelch regulates actin organization via Src64-dependent tyrosine phosphorylation. J. Cell Biol..

[62] Wakabayashi N (2003). Keap1-null mutation leads to postnatal lethality due to constitutive Nrf2 activation. Nat. Genet..

[63] Furlan E (2012). Small population size and extremely low levels of genetic diversity in island populations of the platypus, *Ornithorhynchus anatinus*. Ecol. Evol..

[64] Tinnert J, Hellgren O, Lindberg J, Koch-Schmidt P, Forsman A (2016). Population genetic structure, differentiation, and diversity in *Tetrix subulata* pygmy grasshoppers: roles of population size and immigration. Ecol. Evol..

[65] Harshman LG, Hoffmann AA (2000). Laboratory selection experiments using *Drosophila*: what do they really tell us?. Trends Ecol. Evol..

[66] Mueller, L. D., Rauser, C. L. & Rose, M. R. Population dynamics, life history, and demography in *Advances in Ecological Research: Population Dynamics and Laboratory Ecology* (ed. Robert A. Desharnais) 77–95 (Elsevier Academic Press, 2005).

[67] García-Roger EM, Carmona MJ, Serra M (2017). Modes, mechanisms and evidence of bet hedging in rotifer diapause traits. Hydrobiologia.

[68] Walczyńska A, Franch-Gras L, Serra M (2017). Empirical evidence for fast temperature-dependent body size evolution in rotifers. Hydrobiologia.

[69] Geerts AN (2015). Rapid evolution of thermal tolerance in the water flea. Daphnia. Nat. Clim. Change.

[70] Jansen M (2015). Experimental evolution reveals high insecticide tolerance in *Daphnia* inhabiting farmland ponds. Evol. Appl..

[71] Orsini L, Spanier KI, De Meester L (2012). Genomic signature of natural and anthropogenic stress in wild populations of the waterflea *Daphnia magna*: validation in space, time and experimental evolution. Mol. Ecol..

[72] Boyle EA, Li YI, Pritchard JK (2017). An expanded view of complex traits: from polygenic to omnigenic. Cell.

[73] Collins S (2010). Many possible worlds: expanding the ecological scenarios in experimental evolution. Evol. Biol..

[74] Glaubitz, J. C. *et al*. TASSEL-GBS: A high capacity genotyping by sequencing analysis pipeline. *PLoS one* **9**, e90346 (2014). 10.1371/journal.pone.0090346 PMC3938676 24587335

[75] Li H, Durbin R (2010). Fast and accurate long-read alignment with Burrows-Wheeler transform. Bioinformatics.

[76] Danecek P (2011). The variant call format and VCFtools. Bioinformatics.

[77] Jombart T (2008). Adegenet: a R package for the multivariate analysis of genetic markers. Bioinformatics.

[78] R Development Core Team. *R: A language and environment for statistical computing*. R Foundation for Statistical Computing, Vienna, Austria (2015).

[79] Goudet J (2005). HIERFSTAT, a package for R to compute and test hierarchical F-statistics. Mol. Ecol. Notes.

[80] Goudet J. & Jombart T. Hierfstat: Estimation and tests of hierarchical F‐statistics. Retrieved from, https://CRAN.R-project.org/package=hierfstat (2015).

[81] Lischer HEL, Excoffier L (2012). PGDSpider: An automated data conversion tool for connecting population genetics and genomics programs. Bioinformatics.

[82] Purcell S (2007). PLINK: A tool set for whole-genome association and population-based linkage analyses. Am. J. Hum. Genet..

[83] Foll M, Gaggiotti O (2008). A genome-scan method to identify selected loci appropriate for both dominant and codominant markers: A Bayesian perspective. Genetics.

[84] Pérez-Figueroa A, García-Pereira MJ, Saura M, Rolán-Alvarez E, Caballero A (2010). (2010). Comparing three different methods to detect selective loci using dominant markers. J. Evol. Biol..

[85] De Villemereuil P, Gaggiotti OE (2014). A new FST‐based method to uncover local adaptation using environmental variables. Methods Ecol. Evol..

[86] Narum SR, Hess JE (2011). Comparison of FST outlier tests for SNP loci under selection. Mol. Ecol. Resour..

[87] Nielsen EE (2012). Gene-associated markers provide tools for tackling illegal fishing and false eco-certification. Nat. Commun..

[88] Lotterhos KE, Whitlock MC (2014). Evaluation of demographic history and neutral parameterization on the performance of FST outlier tests. Mol. Ecol..

[89] Quinlan AR, Hall IM (2010). BEDTools: A flexible suite of utilities for comparing genomic features. Bioinformatics.

[90] Conesa A (2005). Blast2GO: A universal annotation and visualization tool in functional genomics research. Application note. Bioinformatics.

