# Supplementary material for: Ecological genomics of adaptation to unpredictability in experimental rotifer populations
Source: Sci Rep. 2019 Dec 23;9:19646. doi: 10.1038/s41598-019-56100-y (PMC6927961; doi:10.1038/s41598-019-56100-y)
Supplement: Supplementary file 1 — Supplementary information [file 41598_2019_56100_MOESM1_ESM.pdf]

Ecological genomics of adaptation to unpredictability in experimental rotifer populations

Eva Tarazona, Christoph Hahn, Lluis Franch-Gras, Eduardo M. García-Roger, María José Carmona & Africa Gómez

Supplementary Table S1: Population means of observed heterozygosity (H_o), expected heterozygosity (H_e), and inbreeding index (F_{IS}) for laboratory and origin populations. P*i*: populations under the predictable selective; U*i*: populations under the unpredictable selective regime. The subindex *i* denotes replicate population within selective regime.

Population	H_o	H_e	F_{IS}
P1	0.22	0.21	-0.03
P2	0.20	0.19	-0.04
P3	0.22	0.20	-0.05
U1	0.21	0.20	-0.04
U2	0.19	0.17	-0.09
U3	0.21	0.20	-0.03
Origin*	0.17	0.21	0.13

*The origin population was composed by clones from nine field populations. Here overall results as a single population are shown (full details for each of field populations in Franch-Gras et al. 2018).

Supplementary Table S2: Summary of candidate SNPs under selection identified by BayeScan using BS1 analysis: (1) Origin population, (2) populations evolved under predictable selective regime, and (3) populations evolved under unpredictable selective regime. The three SNPs identified between two selective regimes (BS2 analysis) are in bold. NA: no functionality found.

SNP_ID	*q*-value	F_{ST}	Gene association
S56713_7995	0.0000	0.3167	Transient receptor potential cation channel subfamily A member
S11659_1691	0.0000	0.2566	---NA---
S11659_1693	0.0000	0.2509	---NA---
S3012_36265	0.0000	0.2402	---NA---
S33929_139	0.0000	0.2340	---NA---
S33929_140	0.0000	0.2333	---NA---
S33929_141	0.0000	0.2330	---NA---
S16309_4100	0.0000	0.2251	cell division cycle
S16309_4101	0.0000	0.2243	cell division cycle
S16309_4103	0.0000	0.2242	cell division cycle

Table S2. (continued)

SNP_ID	*q*-value	F_{ST}	Gene association
S1174_85150	0.0000	0.1965	Chloride intracellular channel exc-4
S13818_736	0.0000	0.1940	---NA---
S13818_738	0.0000	0.1831	---NA---
S2803_16180	0.0000	0.1775	---NA---
S16698_16230	0.0000	0.1617	serine threonine- kinase tousled-like 2 isoform X4
S16698_16228	0.0000	0.1616	serine threonine- kinase tousled-like 2 isoform X4
S16698_16229	0.0000	0.16006	serine threonine- kinase tousled-like 2 isoform X4
S16698_16231	0.0000	0.1592	serine threonine- kinase tousled-like 2 isoform X4
S22481_5630	0.0000	0.1402	---NA---
S17011_30405	0.0000	0.1360	---NA---
S19655_8422	4.0008e-05	0.1352	homeobox prophet of Pit-1-like
S19655_8427	0.0000	0.1346	homeobox prophet of Pit-1-like

Table S2. (continued)

SNP_ID	*q*-value	F_{ST}	Gene association
S19655_8423	0.0000	0.1342	homeobox prophet of Pit-1-like
S34894_4056	0.0000	0.1327	---NA---
S19655_8425	6.2513e-06	0.1324	homeobox prophet of Pit-1-like
S21617_6347	0.0000	0.1305	---NA---
S21617_6352	0.0000	0.1298	---NA---
S21617_6371	0.0000	0.1289	---NA---
S21617_6345	0.0000	0.1271	---NA---
S25654_622	0.0000	0.1197	photoreceptor-specific nuclear receptor-like.
S12176_10377	0.0000	0.1159	regulatory-associated of mTOR isoform X2
**S78024_5745**	0.0001	0.1069	Midasin
S32254_1877	9.7455e-05	0.1042	---NA---

Table S2. (continued)

SNP_ID	*q*-value	F_{ST}	Gene association
S49622_8224	9.7455e-05	0.1023	---NA---
S49622_8242	0.0000	0.1008	---NA---
S49622_8228	4.0008e-05	0.1008	---NA---
S18850_7914	9.7455e-05	0.0998	transcription factor 7-like 2 isoform X8
S5177_8639	0.0033	0.0993	reverse partial 540 F:RNA-directed DNA polymerase activity
S5177_8631	0.0046	0.0992	reverse partial 540 F:RNA-directed DNA polymerase activity
S18850_7920	4.0008e-05	0.0988	transcription factor 7-like 2 isoform X8
S5177_8629	0.0027	0.0982	reverse partial 540 F:RNA-directed DNA polymerase activity
S5177_8630	0.0046	0.0973	reverse partial 540 F:RNA-directed DNA polymerase activity
S23450_12365	9.7455e-05	0.0933	myotubularin-related 13 isoform X5
S18850_7938	0.0001	0.0888	transcription factor 7-like 2 isoform X8
S10378_3420	0.0002	0.0878	pre-mRNA-splicing factor ATP-dependent RNA helicase DHX16

Table S2. (continued)

SNP_ID	*q*-value	F_{ST}	Gene association
S54782_10127	0.0006	0.0856	adenylate cyclase type 2-like isoform X2
S47105_1467	0.015121	0.0851	---NA---
S22890_1785	0.0015407	0.0830	RNA-directed DNA polymerase from mobile element jockey
S18850_8016	0.0009	0.0819	transcription factor 7-like 2 isoform X8
S18850_7974	0.0004	0.0810	transcription factor 7-like 2 isoform X8
S2033_17976	0.0020	0.0807	---NA---
S10770_11251	0.0012	0.0807	---NA---
S12614_9116	0.0053	0.0766	sn1-specific diacylglycerol lipase beta
S49644_27362	0.0272	0.0764	---NA---
S40608_10312	0.0087	0.0762	---NA---
S40608_10343	0.0076	0.0756	---NA---
S49644_27363	0.0375	0.0754	---NA---

Table S2. (continued)

SNP_ID	*q*-value	F_{ST}	Gene association
**S9060_3689**	0.0335	0.0748	RNA-binding single-stranded-interacting 3
**S4644_2726**	0.0060	0.0746	ribosomal S6 kinase alpha-1 isoform X1
S147401_780	0.0068	0.0731	sodium potassium-trasporting ATPase subunit alpha-1
S54782_10014	0.0100	0.0711	adenylate cyclase type 2-like isoform X2
S2597_97237	0.0131	0.0699	---NA---
S3404_8633	0.0171	0.0675	---NA---
S30218_6286	0.0116	0.0674	---NA---
S3312_1846	0.0191	0.0665	Membrane-associated guanylate WW and PDZ domain-containing 2
S4239_2081	0.0251	0.0660	---NA---
S4239_2052	0.0211	0.0659	---NA---
S63942_1714	0.0314	0.0648	liprin-alpha-1-like isoform X3

Table S2. (continued)

SNP_ID	*q*-value	F_{ST}	Gene association
S27662_4458	0.0399	0.0631	---NA---
S8852_22699	0.0231	0.0625	---NA---
S22745_2479	0.0355	0.0622	Condensin complex subunit
S8852_37112	0.0314	0.0618	---NA---
S5457_3425	0.0427	0.0604	---NA---
S11329_34316	0.0456	0.0575	isobutyryl- mitochondrial
S11329_32421	0.0484	0.0575	isobutyryl- mitochondrial

30

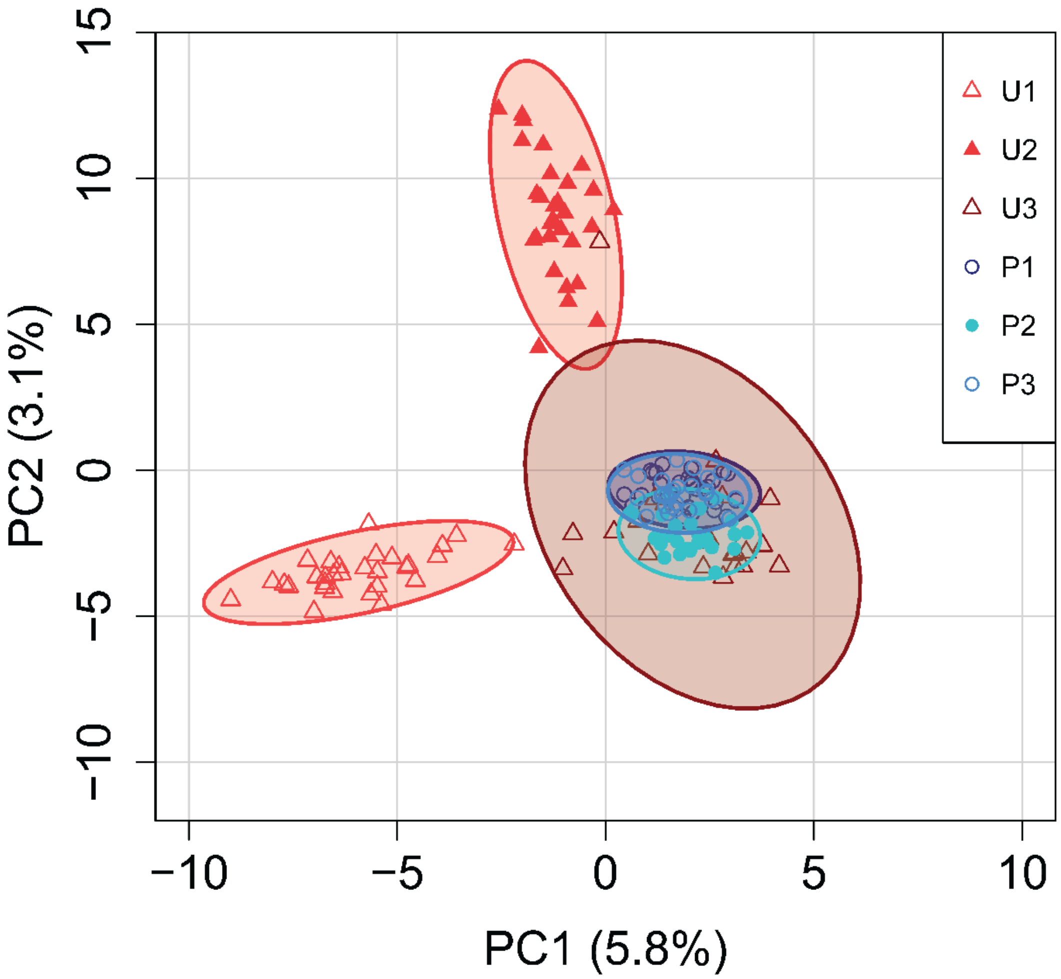

Supplementary Figure S1: Principal component analysis (PCA) plot for the 6,107 SNPs of *Brachionus plicatilis* clones from the six laboratory populations subjected to the two selective regimes (predictable vs unpredictable). Symbols indicate the location of the genotype of each clone in the space defined by the first (PC1; 5.8 % variance explained) and second (PC2; 3.1 % variance explained) principal components. Ellipsoids are the 95% confidence interval the different populations. Color and symbol code: blue circles, populations under predictable regime; red triangles, populations under unpredictable regime.

(A)

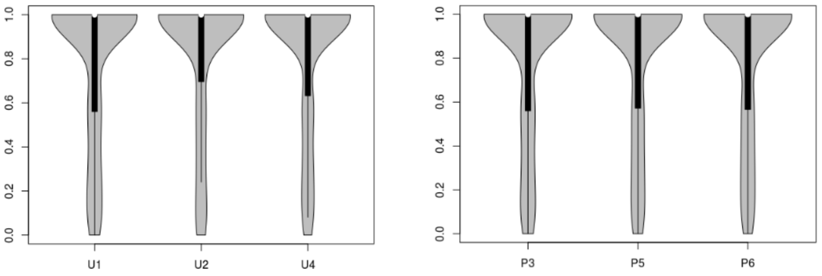

(B)

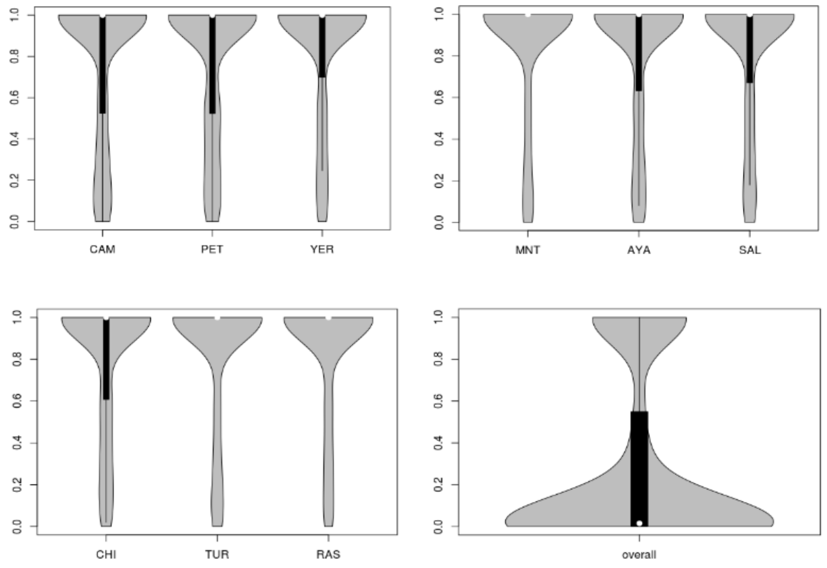

Supplementary Figure S2: Violin plots for results of Hardy-Weinberg (HWE) exact tests of the SNPs of each population. Tests for all populations (x-axis) and probability-values of the exact test (y-axis). (A) Laboratory populations. “U” represents populations under unpredictable regimes and “P” represents populations under predictable regime. (B) Field populations.

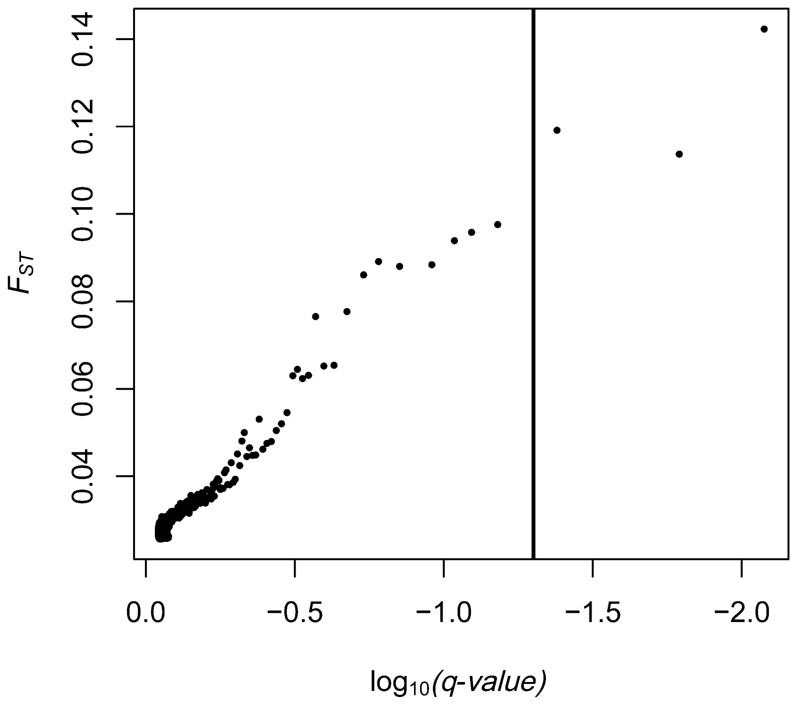

Supplementary Figure S3. Identification of outlier loci putatively under selection in *Brachionus plicatilis* populations using BayeScan analysis for the 6,107 genotyped SNPs. The marker-specific F_{ST} is plotted against the decision factor to determine selection in base-10 log scale $\log_{10}$(*q*-value) using a false discovery rate (FDR) of 0.05. The vertical line is the critical prior odds (PO) of 10 used to identify outlier markers. Markers on the right side of the vertical line are outliers. This analysis (BS2, see main text) was performed using two groupings: (1) populations subjected to the predictable regimen, and (2) populations subjected to the unpredictable one. Each dot represents a SNP.

(A)

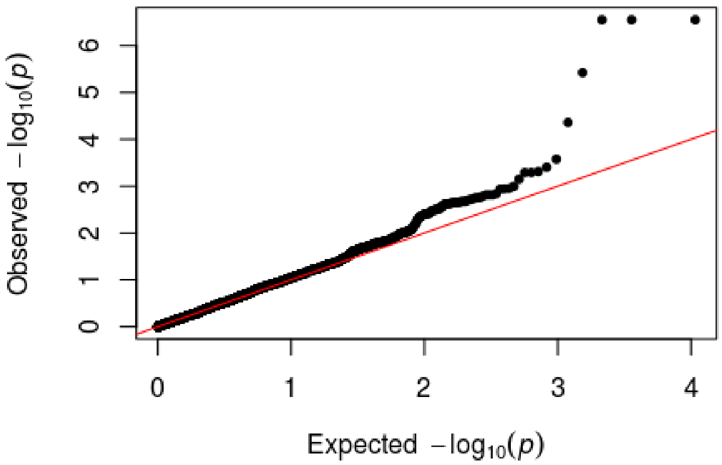

(B)

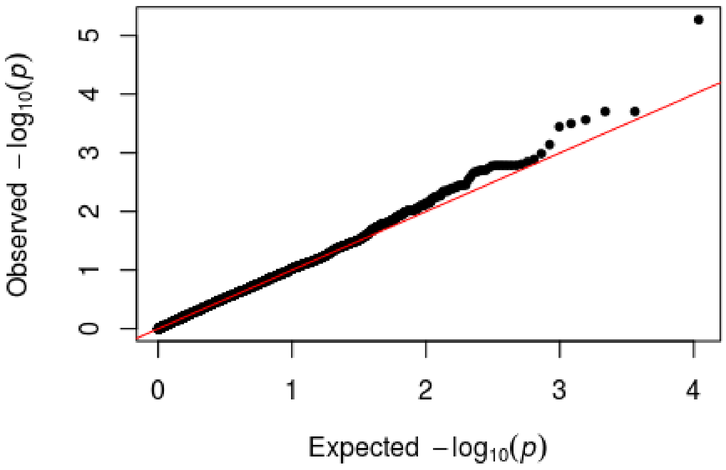

Supplementary Figure S4. Quantile-quantile plot of -log10 (*p*-value) for genotype-phenotype association analyses. (A) Hatching bioassay and (B) timing of sex bioassay. Note that the GWAS analysis was performed despite our data deviating from normality (*p*=0.03 and *p*=0.04, for hatching and timing of sex, respectively).

Supplementary Method S1. Code for genotype-phenotype association analysis using Plink software. This analysis was performed in the clones in which both phenotypic and genotypic data were available (52 and 76 clones for hatching fraction and timing of sex respectively).

```
PLINK v1.90b3w 64-bit
Options in effect:
  --adjust
  --allow-extra-chr
  --allow-no-sex
  --ci 0.95
  --covar ./control_for_population.txt
  --file ../trait_final_plink
  --linear
  --maf 0.01
  --out ./population/assoc_trait_pop
  --pheno ../pheno.txt
```
